# Transcription analysis of the responses of porcine heart to *Erysipelothrix rhusiopathiae*

**DOI:** 10.1371/journal.pone.0185548

**Published:** 2017-10-04

**Authors:** Chao Kang, Qiang Zhang, Weifeng Zhu, Chengzhi Cai, Xiaomei Sun, Meilin Jin

**Affiliations:** 1 Unit of Animal Infectious Diseases, State Key Laboratory of Agricultural Microbiology, Huazhong Agricultural University, Wuhan, Hubei, P.R. China; 2 College of Veterinary Medicine, Huazhong Agricultural University, P.R. China, Wuhan, Hubei, P.R. China; East Carolina University Brody School of Medicine, UNITED STATES

## Abstract

*Erysipelothrix rhusiopathiae* (*E*. *rhusiopathiae*) is the causative agent of swine erysipelas. This microbe has caused great economic losses in China and in other countries. In this study, high-throughput cDNA microarray assays were employed to evaluate the host responses of porcine heart to *E*. *rhusiopathiae* and to gain additional insights into its pathogenesis. A total of 394 DE transcripts were detected in the active virulent *E*. *rhusiopathiae* infection group compared with the PBS group at 4 days post-infection. Moreover, 262 transcripts were upregulated and 132 transcripts were downregulated. Differentially expressed genes were involved in many vital functional classes, including inflammatory and immune responses, signal transduction, apoptosis, transport, protein phosphorylation and dephosphorylation, metabolic processes, chemotaxis, cell adhesion, and innate immune responses. Pathway analysis demonstrated that the most significant pathways were Chemokine signaling pathway, NF-kappa B signaling pathway, TLR pathway, CAMs, systemic lupus erythematosus, chemokine signaling pathway, Cytokine–cytokine receptor interaction, PI3K-Akt signaling pathway, Phagosome, HTLV-I infection, Measles, Rheumatoid arthritis and natural-killer-cell-mediated cytotoxicity. The reliability of our microarray data was verified by performing quantitative real-time PCR. This study is the first to document the response of piglet heart to *E*. *rhusiopathiae* infection. The observed gene expression profile could help screen potential host agents that can reduce the prevalence of *E*. *rhusiopathiae*. The profile might also provide insights into the underlying pathological changes that occur in pigs infected with *E*. *rhusiopathiae*.

## Introduction

*Erysipelothrix rhusiopathiae* is generally regarded as an opportunistic pathogen that causes erysipelas in swine and other diseases in several mammalian and avian species[1]. This pathogen is also the causative agent of erysipeloid, which is a skin disease affecting humans[2]. *E*. *rhusiopathiae* mainly infects hosts via skin scratches or puncture wounds and penetrates the gastrointestinal tract via contaminated water or food intake[3]. In swine, *E*. *rhusiopathiae* is characterized by urticarial diamond-shaped lesions that can quickly progress to an acute septicemic infection or death. An acute infection usually leads to chronic erysipelas that involves self-sustaining, destructive pathological changes in heart valves and joints to induce endocarditis and arthritis, respectively[4]. Nevertheless, the prevention and control of swine erysipelas are often challenging because of its ubiquitous nature and high carrier rate in domestic and wild animals[5]. In China, swine erysipelas has been considered a common pathogenic bacterium since the 1980s. With widespread antibiotic use, this disease has almost disappeared. However, *E*. *rhusiopathiae* outbreak has occurred in the past three decades and has spread domestically in few farms and systemically in provincial areas. Since the recurrence of swine erysipelas as a clinical problem in pig populations, this disease has been classified as a re-emerging disease that has substantially contributed to economic losses in the swine industry.

In a previous study on the pathogenesis of *E*. *rhusiopathiae*, several virulence-associated factors, including a neuraminidase, hyaluronidase, a heat labile capsule, SpaA, and 66–64 kDa antigens, have been successfully identified[4,6,7,8]. However, the molecular mechanisms of *E*. *rhusiopathiae* on phagocytosis or immunity remain poorly understood.

Host–pathogen interactions have been examined in most pathogens to understand their pathogenicity[9,10], but *E*. *rhusiopathiae* has been rarely described. The response mechanism of porcine to *E*. *rhusiopathiae* infection has yet to be fully elucidated. Therefore, sufficient knowledge on the responses to *E*. *rhusiopathiae* infection in pigs should be obtained to enhance our understanding of this infection. This study aimed to perform a whole-genomic analysis of the transcriptional responses of a pig heart to virulent and avirulent strains and PBS by using Affymetrix Porcine Gene 1.0 ST Microarray, which contains 394,580 probes and 19,212 gene-level probe sets, and to elucidate the immune responses of hosts to *E*. *rhusiopathiae*.

## Material and methods

### Bacterial strains

In 2013, *E*. *rhusiopathiae* strain SE38 showing high virulence to pigs was isolated from the heart of a diseased piglet in Hubei Province, China. *E*. *rhusiopathiae* strain G4T10, which is an attenuated strain, did not elicit evident virulence to pigs.

### Animal infection and tissue collection

Experimental protocols were approved by the Laboratory Animal Monitoring Committee of Hubei Province and performed accordingly.

At 45 days of age, nine piglets obtained from a commercial herd free of *E*. *rhusiopathiae* disease were subjected to controlled laboratory conditions. These piglets were randomly divided into three groups with three individuals per group. Two groups of piglets were challenged intradermally with 5 × 10^8^ colony-forming units of SE38 strain and G4T10 strains, respectively, and the remaining piglets were used as controls challenged by an identical volume of PBS. Bacterial concentrations were determined through plate dilution on inoculum samples pre- and post-challenge. The administered doses were confirmed, and the viability of the challenge strains was retained during the challenge. The pigs were slaughtered at 4 days post-infection (dpi). Organs, namely, heart, lung, liver, spleen, kidney, and brain, of the pigs were examined through bacterial isolation and PCR. Heart samples were aseptically collected and immediately frozen in liquid nitrogen for subsequent RNA isolation. The remaining heart specimens from the SE38-infected pigs, G4T10-infected pigs and control pigs were fixed in 4% neutral buffered formalin, embedded in paraffin, and stained with hematoxylin and eosin (HE) to investigate their pathological damage.

### RNA preparation for microarray experiment

RNA was extracted from approximately 200 mg of each sample and purified by using an RNeasy mini kit (QIAGEN) following the manufacturer’s instruction. The integrity and quantity of RNA were assessed using an Agilent Bioanalyzer 2100. The quality of RNA was checked with formaldehyde denaturing gel electrophoresis in 1.2% agarose gels, which showed dispersed bands (28S and 18S) without any evident smearing patterns that would indicate degradation.

### Microarray hybridization and data analyses

RNA labeling and hybridization were conducted via a commercial Affymetrix array service. A 2 μg aliquot of total RNA was converted to double-stranded cDNA with a one-cycle cDNA synthesis kit (Affymetrix), and biotin-tagged cRNA was produced with an Affymetrix WT amplification kit and a GeneChip WT terminal labeling kit. The resulting biotagged cRNA was fragmented into strands of 35–200 bases in length in accordance with Affymetrix’s protocols. These strands were then hybridized to GeneChip Porcine Genome 1.0 ST Array. Hybridization was performed at 45°C with rotation for 16 h. GeneChip arrays were washed, stained with streptavidin phycoerythrin on an Affymetrix Fluidics Station 450, and scanned on GeneChip Scanner 3000. Nine microarrays corresponding to the RNAs from the hearts of three SE38-infected piglets and two other groups were used in the experiment.

Hybridization data were analyzed using GeneChip Operating software (GCOS 1.4). Raw expression data were normalized through robust multiarray averaging (RMA) with quantile normalization and then imported into GeneSpring 12.5 to detect the transcripts exhibiting consistent changes within the triplicates and the transcripts showing differential expression. These data were also analyzed through unpaired t-test to determine the differentially expressed transcripts in the three groups. Differentially expressed (DE) genes were identified on the basis of the changes in p-value calculated by 0.05 and the two-fold change as an empirical criterion among the highly pathogenic, avirulent strain, and PBS groups. The DE genes were subjected to hierarchical clustering (Ver.3.0) and TreeView (Ver.1.1.1) analysis. The genes with significant similarities to the transcripts in the nr database based on BLASTX searches were selected for Gene Ontology (GO) analysis with DAVID annotation (http://david.abcc.ncifcrf.gov/home.jsp). Annotation results were obtained by inputting a list of gene symbols as identifiers. The mRNA accession numbers were deposited to the National Center for Biotechnology Information (NCBI) and the latest annotation was obtained. Functional pathways (Kyoto Encyclopedia of Genes and Genomes [KEGG]) were examined by using the online DAVID analysis system. The DE genes in the porcine infected with SE38 or PBS were analyzed using STRING, a database of known and predicted protein interactions. Our microarray results were deposited to NCBI’S Gene Expression Omnibus (GEO) database, and the accession numbers are as follows: GSM2425704, GSM2425705, GSM2425706, GSM2425707, GSM2425708, GSM2425709, GSM2425710, GSM2425711, GSM2425713.

### Quantitative reverse-transcription PCR (qRT-PCR) analysis

qRT-PCR was performed to validate the data selected from the microarray experiments with SYBR green and to monitor the expression of a subset of genes over time.

Total RNA was extracted from the hearts of each group by using TRIzol, and 5 μg was included as a template for first-strand cDNA synthesis in a Superscript II cDNA amplification system (Invitrogen) in accordance with the manufacturer’s instructions. Glyceraldehyde-3-phosphate dehydrogenase(GAPDH) was used as an endogenous control. The specific primers used in the qRT-PCR assays are listed in Table 1. qRT-PCR was conducted in triplicate for all of the reactions by using a SYBR green detection system and an ABI 7900 HT sequence detection system. Relative standard curves for target and endogenous control primer pairs were obtained to verify comparable PCR efficiencies. Afterward, comparative (2^-ΔΔ^) Ct method was applied. Reaction specificity was confirmed by performing the Melt procedure at the end of amplification.

**Table 1:** Validation of microarray by qPCR.

Gene	Primer sequence 5’-3’	Accession	regulation	Microarray fold change	qPCR fold change
CCL2	F:GCAAGTGTCCTAAAGAAGCAGTGA	NM_214214	UP	6.5181713	14.8311899
	R:TGCTTGGGTTCTGCACAGATC				
TLR4	F:ACTTCCAGGTGGATGTTTCGA	NM_001113039	UP	3.7690496	8.382051197
	R:TTGATGCGCTAAGAGTTGAATTG				
TLR7	F:TTACCAGGGCAGCCAGTTCT	NM_001097434	UP	2.9303472	8.973114925
	R:TTGCATACTTGTCTGTCATCACAAA				
MYD88	F:ATCCTGCGGTTCATCACTGTCT	NM_001099923	UP	3.232283	6.330957307
	R:CAAGGCGAGTCCAGAACCA				
CD86	F:CACGGAACTCTACAATGTATCAATCA	NM_214222	UP	2.867575	7.23
	R:GACACAGACGATGCTCACATTTG				
CXCL10	F: CTGGCCCTGCTACTGACACT	NM_001008691	UP	11.846697	32.82090997
	R: GTCCCAAAGTGGAATGATCC				
HBB	F: GCTCCTGGGCAACGTGATAG	AK236946	down	11.830982	87.43645471
	R: CACATCCGGGTTGAAGTCATG				
CSRP3	F: CAAGACCTGTTTTCGCTGTG	NM_001172368	down	3.3533556	1.470776809
	R: CCAATACCTGTAGGGCCAAA				
ADRA1A	F: CGATGGAGTCTGTGAATGGAAA	NM_001123072	down	2.4406312	3.8621578
	R: TCTTTGGGCACCGTAATCCT				
GAPDH	F: TGCCGCCTGGAGAAACCT	XR_002343817	endogenous control		
	R: GCATCAAAAGTGGAAGAGTGAGTG				

## Results

### Clinical evaluation of infected pigs

**Fig 1:**
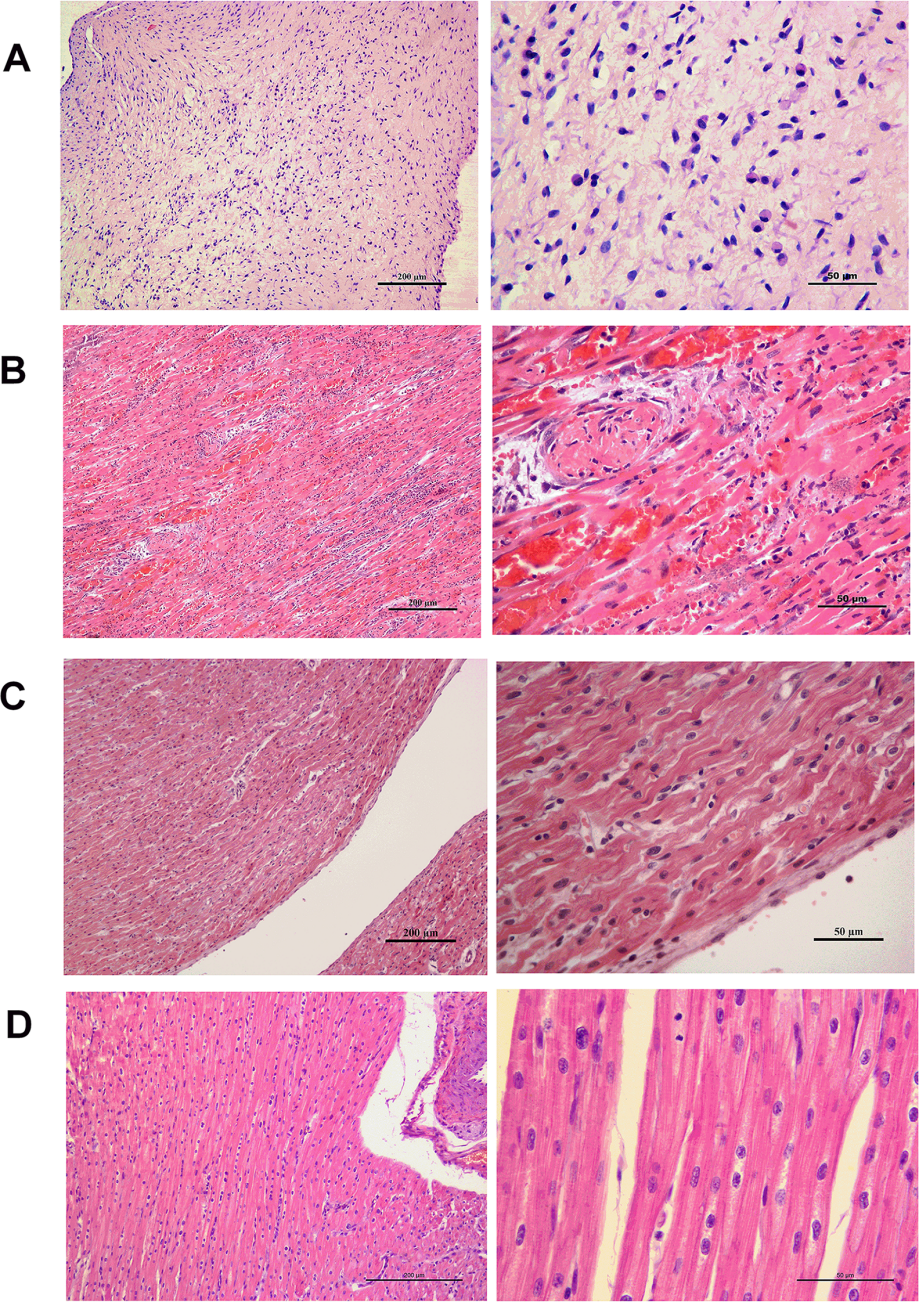
Representative histopathological photomicrograph of heart lesions in pigs infected with *E*. *rhusiopathiae* strain SE38, G4T10 and control. (A) Heart of a SE38-infected pig with endocarditis and neutrophil infiltration. (B) Thrombogenesis, myocardial necrosis, and inflammatory cell infiltration. (C) Heart of a G4T10-infected pig at 200 and 50μm. (D) PBS control at 200 and 50 μm.

The clinical signs and lesions of the *E*. *rhusiopathiae* disease were observed in the group inoculated with SE38, whereas no clinical signs were detected in the pigs inoculated with G4T10 or PBS. In the group challenged with SE38, two pigs manifested larger cutaneous lesions and diamond skin lesions appeared all over the body at 2 and 3 dpi, respectively. Concurrently, the remaining pig retained small skin lesions. These three pigs were found unconscious, with apocleisis and pyrexia (40°C–41°C). In the control group, three pigs remained clinically normal throughout the experiment and did not possess lesions. *E*. *rhusiopathiae* detected through bacterial isolation and PCR in different samples is shown in Table 2. The results indicated that *E*. *rhusiopathiae* could be detected in the heart of the three pigs challenged with *E*. *rhusiopathiae* strain SE38 but not in the brain. By contrast, the pigs inoculated with G4T10 or PBS could not be observed in either heart or brain. In a macroscopic view, the heart tissues from the SE38 group barely showed apparent damage. The heart tissues of the SE38-infected pigs manifested thrombus, endocarditis, myocardial necrosis, dilated intercellular spaces, and a large amount of infiltrating inflammatory cells. Conversely, the PBS- and G4T10-administered pigs did not show heart lesions(Fig 1).

**Table 2:** Results of culture and PCR analysis for three pigs challenged with *E*. *rhusiopathiae* presented as the number of positive pigs/total pig samples.

Samples	*E*.*rhusiopathiae* culture			PCR		
	SE38 group	G4T10 group	PBS group	SE38 group	G4T10 group	PBS group
Heart	3/3	0/3	0/3	3/3	0/3	0/3
Liver	1/3	0/3	0/3	2/3	0/3	0/3
Spleen	1/3	0/3	0/3	2/3	0/3	0/3
Lung	1/3	0/3	0/3	2/3	0/3	0/3
Kidney	3/3	0/3	0/3	3/3	0/3	0/3
Brain	0/3	0/3	0/3	0/3	0/3	0/3
Total site	9/18			12/18		
%positive	50			67.7		

### Gene expression alterations in heart tissues

**Fig 2:**
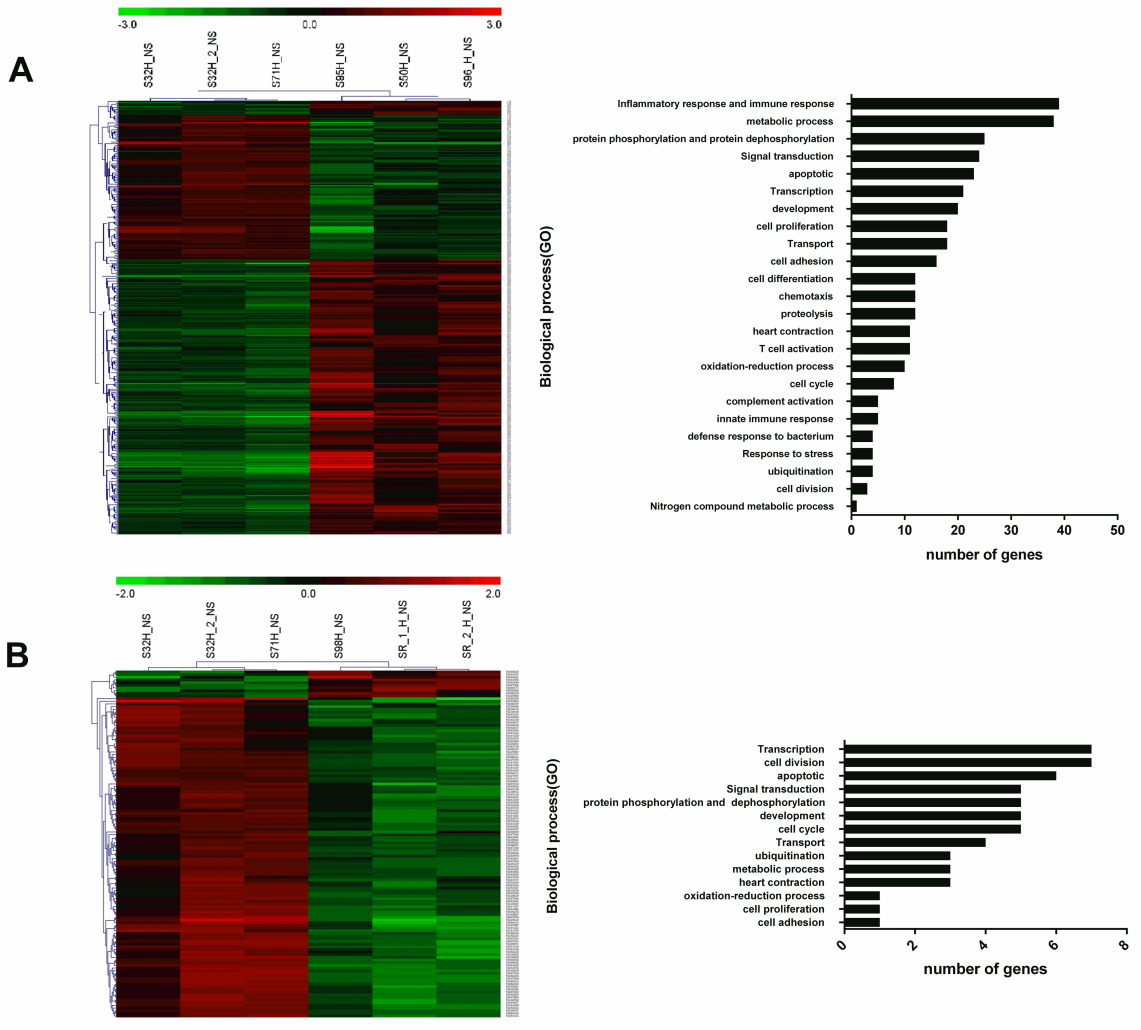
Clustering and characterization of the differential expression of genes. (A) SE38 group vs. PBS group. S95H_NS, S50H_NS and S96_H_NS belong to SE38 group; S32H_NS, S32H_2_NS and S71H_NS belong to PBS group. DE genes that showed clear functional annotation at 4 dpi between the SE38 and PBS groups were selected for cluster analysis as described in methods. At 4 dpi, a set of 262 genes were upregulated and the remaining 132 genes were downregulated. (B) G4T10 group vs. PBS group. S98H_NS, SR_1_H_NS and SR_2_H_NS belong to G4T10 group; S32H_NS, S32H_2_NS and S71H_NS belong to PBS group. Each row represents a separate gene and each column represents a separate piglet. Red indicates the increased gene expression levels; green denotes the decreased levels compared with normal samples.

To analyze the pathogenesis of *E*. *rhusiopathiae*, we challenged experimental pigs with *E*. *rhusiopathiae*. The DE gene expression profile of these hearts was determined. The genes with relative transcription levels of fold change (FC) ≥ 2 and p ≤ 0.05 were upregulated, and those with FC ≤ 0.5 and p ≤ 0.05 were downregulated. A total of 394 DE transcripts (p ≤ 0.05) were detected in the group with active *E*. *rhusiopathiae* infection compared with the group administered with PBS at 4 dpi (Fig 2A). Among these transcripts, 262 were upregulated and 132 were downregulated. Few transcripts were significantly and differentially expressed when the infected group was compared with the G4T10 and PBS groups. In particular, only 10 transcripts were upregulated and 120 transcripts were downregulated (Fig 2B).

### Validation of microarray data by quantitative real-time PCR (qPCR)

qPCR analysis was performed to validate the statistical significance of the DE genes identified from microarray experiments. Six remarkably upregulated genes and three downregulated genes were selected for qRT-PCR analysis and amplified from the SE38-infected and control samples. The expression level of the selected upregulated genes was higher in the SE38-infected samples than in the control samples (Table 1). This result indicated that the variation trends of these nine selected genes were consistent in qRT-PCR and microarray. Therefore, the microarray analysis performed in this study was reliable.

### Analysis of DE genes by gene ontology(GO)

Of the 394 unique DE transcripts, 272 could be determined on the basis of BLASTX searches and annotated with DAVID or a search in the GenBank database. Among these transcripts, 199 unique genes were obtained and grouped into 24 categories based on biological process GO terms or potential biological process classification reported in recent publications. At 4 dpi, the DE genes were mainly clustered into the following functional groups: inflammatory response and immune response (e.g., TNF, TLR4, CCL2, CCL4, CXCL10, and MYD88); signal transduction (e.g., STAT2, CD48, TLR7, ARHGAP30, FCER1G, and TNFRSF11B); apoptosis (e.g., CASP1, CTSH, STAT1, and JAK3); transport (e.g., HBB, SCN7A, KCNA5, TCN1, and SLC15A3); protein phosphorylation and protein dephosphorylation; metabolic process; chemotaxis; cell adhesion; and innate immune response (Fig 2A).

The upregulated expression of these genes associated with inflammatory response and immune response suggested the occure of an inflammatory response in *E*. *rhusiopathiae* infection. The pathological heart section also revealed the inflammatory and immune responses of the pigs. In these genes, CXCL10 was upregulated by more than tenfold. Five genes, namely, IGJ, C3, CCL2, CCL23, and CD180, were upregulated by more than fivefold.

Cell adhesion molecules (CAMs) are implicated in the regulation of various fundamental cellular processes. In this study, CAMs can be further divided into the following three groups on the basis of the GO analysis of the microarray data: IgSF CAMs (ICAM-1 and vascular cell adhesion molecule-1 [VCAM-1]), integrins (ITGB6 and ITGAL), and selectins (SELE and SELL). ICAM-1, VCAM-1, ITGAL, SELE and SELL are upregulated, and ITGB6 is downregulated.

In the G4T10 and PBS groups, 58 DE transcripts could be determined through BLASTX search and DAVID annotations or GenBank search. These DE transcripts were grouped into 14 categories based on biological process. No DE transcript was found responsible for inflammatory and immune responses. These DE transcripts mainly focused on transcription, cell division, and apoptosis (Fig 2B).

### Pathway analysis

The DE genes were subjected to pathway mapping according to DAVID database to gain insights into different biological processes associated with *E*. *rhusiopathiae* infection, and *Sus scrofa* was selected as our background. At 4 dpi, the predominant pathways included the Chemokine signaling pathway, NF-kappa B signaling pathway, TLR pathway, CAMs, Cytokine–cytokine receptor interaction, PI3K-Akt signaling pathway, Phagosome, HTLV-I infection, Measles, Rheumatoid arthritis, systemic lupus erythematosus, and natural-killer-cell-mediated cytotoxicity. The TLR signaling pathway plays a major role in SE38 *E*. *rhusiopathiae* infection. These pathway-related genes significantly differ in the G4T10 group. These results also suggested that the host could activate its immune and inflammatory responses against a pathogenic infection through different strategies at 4 dpi (Table 3).

**Table 3:** DE genes analysis base on KEGG in SE38 and PBS group.

pathway name	Number	Gene
Chemokine signaling pathway	15	CCL2, LYN, CCR1, CCL8, CCL19, STAT1, CCL4, VAV1, STAT3, STAT2, CXCL10, PLCB4, CCL23, GNG10, JAK3
NF-kappa B signaling pathway	14	BTK,CCL19,CCL4,CD14,CD40,LYN,ICAM-1,LY96,MYD88,TLR4,TNF,TNFAIP3,TNFSF13B,VCAM1
Toll-like receptor signaling pathway	14	CCL4,CD14,CD40,CD80,CD86,CXCL10,MAP3K8,IRF5_tv1,LY96,MYD88,SPP1,TLR4,TLR7,TNF
Cytokine-cytokine receptor interaction	13	TNF, CCL2, OSMR, CCR1, CCL8, CCL19, CD40, CCL4, CXCL10, TNFRSF11B, PRLR, TNFSF13B, IL2RG
PI3K-Akt signaling pathway	12	PRLR, OSMR, TNC, GNG10, ITGB6, PIK3AP1, IL2RG, TLR4, ANGPT1, ITGA4, JAK3, SPP1
Phagosome	11	MRC1, MSR1, NCF2, TUBB2B, FCGR2B, C3, TAP1, TLR4, CTSS, GP91-PHOX, CD14
HTLV-I infection	11	VCAM1, ITGAL, EGR2, TNF, ETS2, SPI1, IL2RG, CDC20, JAK3, PTTG1, CD40
Cell adhesion molecules (CAMs)	10	CD40,CD80,CD86,ICAM-1,ITGA4,ITGAL,PTPRC,PVR, SELL,VCAM1
Measles	10	MYD88, FCGR2B, IL2RG, TLR4, JAK3, TNFAIP3, STAT1, TLR7, STAT3, STAT2
Rheumatoid arthritis	9	ANGPT1,CCL2,CD80,CD86,ICAM-1,ITGAL,TLR4,TNF, TNFSF13B
Staphylococcus aureus infection	8	C1QA,C1R,C4,CFB,FCGR2B,ICAM-1,ITGAL,MASP1
Natural killer cell mediated cytotoxicity	8	CD48, ITGAL, TNF, FCER1G, VAV1, TYROBP, PRKCB, LCP2
Complement and coagulation cascades	7	C1QA,C1R,C4,CFB,PLAUR,F13A1,MASP1
Systemic lupus erythematosus	7	C1QA,C1R,C4,CD40,CD80,CD86,TNF

### STRING analysis of the relationships between DE genes

**Fig 3:**
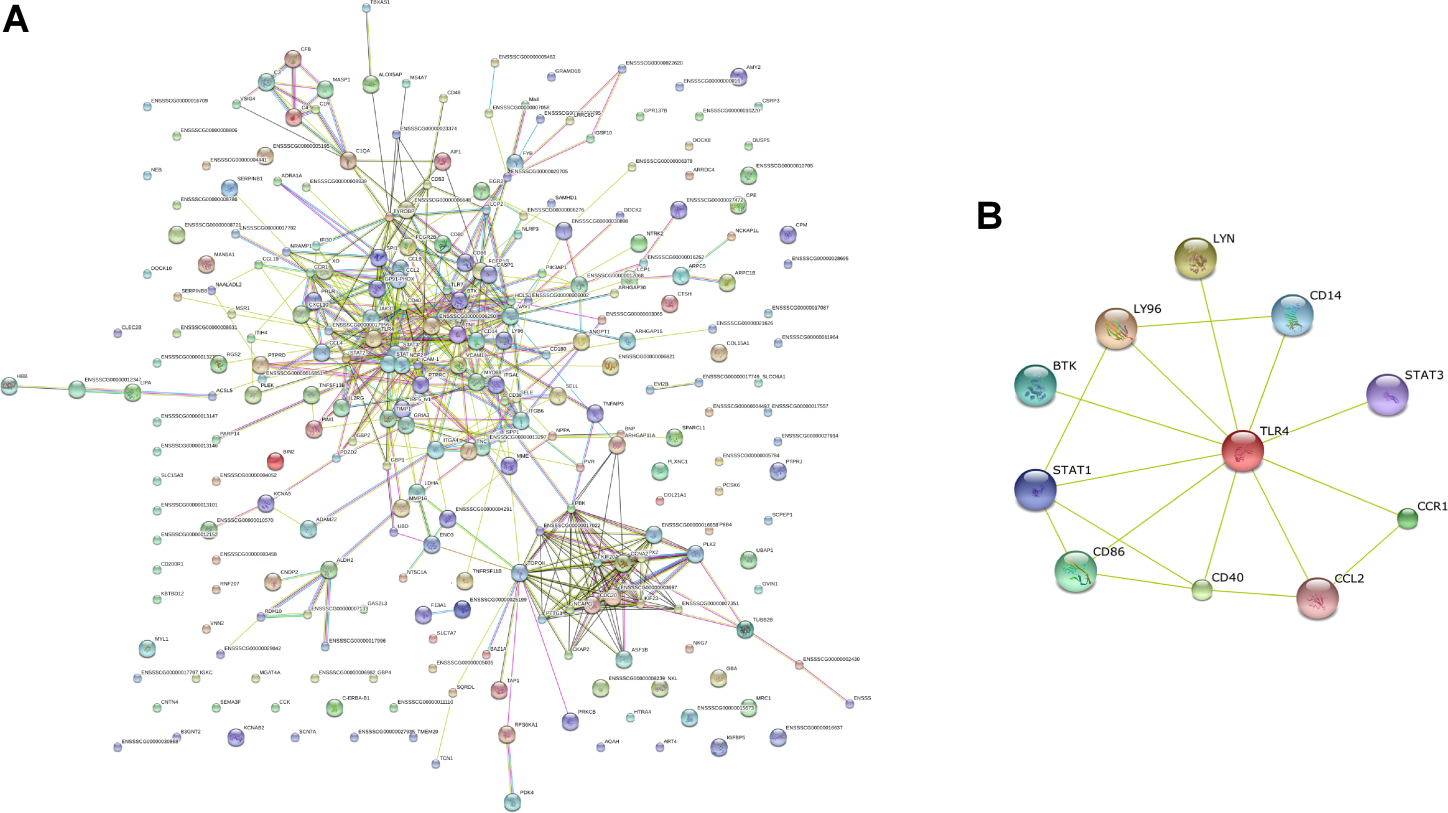
STRING analysis of the relationship between DE genes. (A) the DE genes in piglets infected ER were analyzed using STRING database. (B) the network of DE genes related to TLR4.

STRING is a web-based interface that can predict protein associations with direct binding or indirect interaction, such as participation in the same metabolic pathway or cellular process, through genomic context, high-throughput experiments, co-expression, and literature data (http://string.embl.de). In our study, the DE genes were analyzed by using STRING to predict the network of the proteins encoded by these genes. Among the 394 annotated DE genes, 280, including 195 upregulated genes and 85 downregulated genes, were eligible for STRING analyses when *Sus scrofa* database was chosen. A combined score of 0.400 was selected to search for the possible associations among the DE genes. The network of the predicted associations for the DE-gene-encoded proteins are shown in Fig 3A. STRING analysis demonstrated that some molecules were key to linking with other proteins. The results indicated that genes CCL4, CD14, CD40, LY96, MYD88, TLR4, TLR7,TNF belong to many signaling pathways and other inflammatory or immune response. However, many of the proteins were not linked to other proteins. Thus, their functions were unrelated or unknown. In Fig 3B, 10 proteins encoded by the upregulated DE genes were associated with the TLR4 signaling pathway according to texting evidence.

## Discussion

Swine erysipelas was widespread in China 20 years ago but was subsequently controlled because of vaccine and antibiotic use. However, *E*. *rhusiopathiae* infection has recurred as a serious clinical problem in pig populations in China. The molecular mechanisms of host factors involved in the pathogenesis of *E*. *rhusiopathiae* infection have yet to be established because of the lack of comprehensive information about host responses. A key symptom caused by *E*. *rhusiopathiae* infection involves destructive pathological changes in heart valves and endocarditis[11]. Hence, a genomic expression analysis was performed in our study to obtain global information regarding host responses in the heart of infected pigs. This study is the first to report the use of GeneChip Porcine Genome Array for the investigation of transcriptional responses to *E*. *rhusiopathiae* infection. Our clinical and pathological findings were consistent with previous results[5]. Nevertheless, our unique immunological data help explain the temporary clinical signs and severe pathological changes associated with the disease. GO, KEGG-pathway, and STRING analyses suggested that these DE genes belonged to various functional categories and signal pathways.

A larger set of DE genes from the pathogenic strain was involved in inflammatory and immune responses, whereas the profiles of the avirulent strains were similar to those of the PBS-treated samples. Further analysis revealed that these DE transcripts were upregulated genes. Therefore, these DE genes were implicated in *E*. *rhusiopathiae* infection.

TLRs belong to a family of pattern-recognition receptors that recognize conserved motifs in pathogens[12] and play an essential role in innate immune responses. TLRs have also been recognized as a link among innate immune system, inflammation, and atherosclerosis[13].

TLR4, the most widely examined TLR, is a member of the TLR family and the receptor complex that mediates hyper-inflammatory response and contributes to high mortality during endotoxin shock and severe sepsis[14,15]. TLR4 is ubiquitously expressed in cardiovascular cells; TLR4 signaling is also associated with vascular inflammatory pathologies, such as atherosclerosis[16]. Through TLR4, platelets act as inflammatory sentinels that surround and isolate an infection but modulate pro-inflammatory cytokine release. Thus, platelet TLR4 is implicated in sepsis because of these events[17,18]. Coagulation abnormalities are commonly observed in severe sepsis, and the presence of TLR4 on platelets can be considered a link between disseminated intravascular coagulation and sepsis[19]. Our data showed that TLR4 was expressed most abundantly in the heart and was upregulated by 3.76-fold. HE staining demonstrated that many inflammatory cytokines were released and thrombosis occurred. This result implied that TLR4 might function in pathogen recognition and innate immunity activation and consequently induce a thrombotic tendency.

CD14 upregulated by 6.24-fold is another bacterial receptor that cooperates with TLR4 via MYD88 and possibly triggers inflammatory responses[20]. CD14 acts as a co-receptor with TLR 4 and MD-2 in the detection of bacterial lipopolysaccharide (LPS) or lipoteichoic acid[21]. Ly96 upregulated by 5.00-fold is also known as “MD2” likely associated with TLR4 on cell surfaces and implicated in mediating signal-transduction events induced by LPS, which is found in most Gram-negative bacteria[22]. MyD88 upregulated by 3.23-fold is a universal adapter protein used by almost all TLRs except TLR3 to activate the transcription factor NF-κB, cytokine secretion, and inflammatory response. These findings implied that *E*. *rhusiopathiae* might have developed sophisticated strategies to activate the TLR4 signaling that occur via MyD88 for the course of host cells infected. The activation of TLR4 pathway may be associated with coagulopathy. Nevertheless, the exact mechanisms of *E*. *rhusiopathiae* in modulating the TLR4 pathway should be further investigated.

TLR7 upregulated by 2.93-fold is associated with lupus syndrome and involved in mediating interferon-I production stimulated by the transcription factors IRF5 (upregulated 2.11-fold) and IRF7[23]. KEGG analysis revealed the systemic lupus erythematosus pathway involving CD86, TNF, C4, FCGR2B, CD80, C3, CD40, and C1S. We observed skin symptoms similar to those of the lupus syndrome in the piglet infected with *E*. *rhusiopathiae*. TLR7 may function in the formation of diamond-shaped skin lesions during *E*. *rhusiopathiae* infection.

Chemokines comprise a superfamily of polypeptides with a wide range of activities that include recruitment of immune cells to sites of infection and inflammation[24]. In this study, the chemokines CCL2, CCL4, CCL8, CCL19, CCL23, and CXCL10 were upregulated. CCL2 is known as monocyte chemotactic protein 1 (MCP1) upregulated by more than 5-fold. CCL2 is implicated in the pathogenesis of several diseases, such as psoriasis, rheumatoid arthritis, and atherosclerosis, which are characterized by monocytic infiltrates[25]. CCL4 belongs to the CC chemokine family and known as macrophage inflammatory protein-β[26]. CCL4 and CCL3 are major factors produced by macrophages after they are stimulated with bacterial endotoxins. These factors are crucial for immune responses to infection and inflammation[27]. CXCL10 is a small cytokine that belongs to the CXC chemokine family and attributed to chemoattraction for monocytes/macrophages, T cells, and natural killer cells and inhibition of bone marrow colony formation and angiogenesis[28,29]. TNF is a key cytokine that stimulates inflammatory cell responses[30]. Our study showed that *E*. *rhusiopathiae* could induce TNF expression in piglets at 4 dpi. These results suggested that *E*. *rhusiopathiae* infection modulated the immune responses of pigs by inducing cytokine production and promoting inflammatory responses.

CAMs and their ligands are necessary to regulate lymphocyte recirculation and leukocyte emigration into an inflamed or injured tissue[31]. We discovered several cell adhesion-related DE genes, including VCAM-1, ITGAL, CD86, CD80, SELL, ICAM-1, TNC and SELE, during *E*. *rhusiopathiae* infection. ICAM-1 and VCAM-1 are essential adhesion molecules involved in local inflammatory responses occurring in vascular walls[32]; for instance, circulating leukocytes are recruited to the vascular endothelium and they further migrate into subendothelial spaces during atherosclerosis, which is an inflammatory disease[33]. In acute and chronic inflammatory diseases, endothelial cells are activated and express high ICAM-1 levels, in addition to VCAM-1and SELE. VCAM-1 mediates the adhesion of lymphocytes, monocytes, eosinophils, and basophils to the vascular endothelium. This protein also functions in leukocyte–endothelial cell signal transduction and possibly participates in atherosclerotic development. During inflammation, SELE and SELL are expressed by different tissues and involved in recruiting leukocytes to injury sites[34]. These CAMs may also participate in the pathogenicity of *E*. *rhusiopathiae*.

In summary, this study is the first to evaluate the gene expression profile of *E*. *rhusiopathiae*-infected pigs. Microarray analysis showed that the expression levels of more than 300 genes (FC > 2.0 or FC<0.5 and p-value< 0.05) were altered after the pigs were infected with *E*. *rhusiopathiae*. These DE genes were involved in inflammatory and immune responses, signal transduction, CAMs, apoptosis and other processes. *E*. *rhusiopathiae* could induce cytokines mainly via the TLR4 pathway, and high levels of cytokines and toxins secreted by bacteria could eventually destroy deep tissues and cause septicemia. Consequently, coagulopathy could occur.

## Supporting information

S1 File Complete list of DE transcripts compared SE38(virulent) group to PBS group(FC≥ 2 or FC ≤ 0.5 and p ≤ 0.05). (XLSX)

S2 File Complete list of DE transcripts compared G4T10(avirulent) group to PBS group(FC≥ 2 or FC ≤ 0.5 and p ≤ 0.05). (XLSX)
